# An update on emergency care and emergency medicine in Russia

**DOI:** 10.1186/s12245-015-0092-1

**Published:** 2015-11-25

**Authors:** Anthony Rodigin

**Affiliations:** Department of Emergency Medicine, Sutter Delta Medical Center, 3901 Lone Tree Way, Antioch, CA 94509 USA

**Keywords:** Russian Federation, Russia, Emergency care, Emergency medicine, Ambulance, International medicine

## Abstract

Russia’s national healthcare system is undergoing significant changes. Those changes which affect healthcare financing are particularly vital. As has often been the case in other nations, the emergency care field is at the forefront of such reforms. The ongoing challenges constitute the environment in which the hospital-based specialty of emergency medicine needs to develop as part of a larger system. Emergency care has to evolve in order to match true needs of the population existing today. New federal regulations recently adopted have recognized emergency departments as the new in-hospital component of emergency care, providing the long-needed legal foundation upon which the new specialty can advance. General knowledge of Western-style emergency departments in terms of their basic setup and function has been widespread among Russia’s medical professionals for some time. Several emergency departments are functioning in select regions as pilots. Preliminary data stemming from their operation have supported a positive effect on efficiency of hospital bed utilization and on appropriate use of specialists and specialized hospital departments. In the pre-hospital domain, there has been a reduction of specialized ambulance types and of the number of physicians staffing all ambulances in favor of midlevel providers. Still, a debate continues at all levels of the medical hierarchy regarding the correct future path for emergency care in Russia with regard to adaptation and sustainability of any foreign models in the context of the country’s unique national features.

## Background

**Fig. 1:**
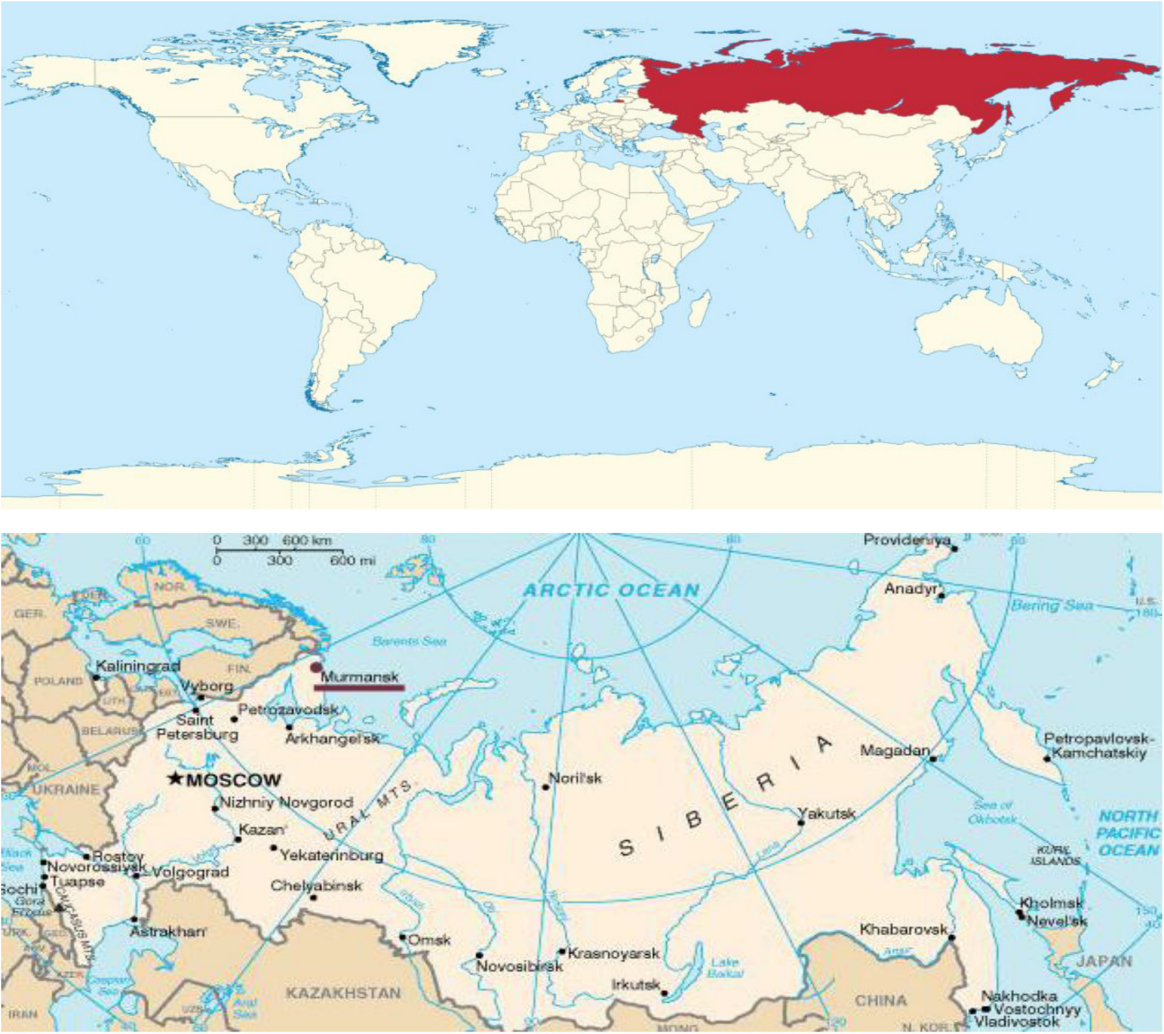
Russian Federation—location on the world map and the surrounding states

Very few articles describing the state of emergency medicine and emergency care in Russia are available in English [1, 2]. This paper reviews select topics pertaining to prospects for emergency medicine development in the Russian Federation (Fig. 1). It aims to generate further interest on the subject among providers who practice emergency medicine or are otherwise involved in emergency care in other nations.

## Discussion

### Private sector versus public sector

No different from many other nations, the private sector of Russian healthcare exists in parallel with the government-run national system which predominates. What is less typical is the lack of any significant or uniform integration between the two components in terms of emergency care services. For instance, while private ambulance companies have been plentiful [3] since the 1990s (working for upfront or pre-arranged fees, while contracted with similarly private hospitals and clinics), such enterprises are found largely in the cities and, more importantly, are affordable for a small segment of the population. In addition, these companies do not participate in the public emergency ambulance dispatch telephone networks (“03” or “103”). Thus, government-controlled services, organized and funded at the federal level, continue to constitute the bulk of emergency care available to the average person—in principle, guaranteed to all citizens free of charge [4].

### Outsourcing and for-pay EMS divisions

In some areas of the country, for instance the cities of Perm, Kirov, Ufa, and a few others (Western Russia), contract-based government outsourcing of ambulance services has recently been attempted with variable success [5]. However, such experimental handovers of operational ownership so far have not included the actual ambulance personnel. Ambulance crew members have remained government employees now staffing ambulances owned, supplied with equipment and maintained by private entities.

As a somewhat different approach, for some years many official EMS services at the municipal level have been running their so-called for-pay divisions, providing alternatives to using mainstream “03” crews for various needs (for instance, medical support for public events), albeit no longer for free [6]. While for-pay services generate funds for local agencies, it has been controversial and a matter of debate whether such fee-for-service options and add-ons ought to remain a part of the government’s emergency ambulance care specifically.

### Volunteer workforce

Unlike countries where volunteers make up a substantial component of healthcare organizations and services, volunteerism is neither common nor popular in today’s Russia. Undoubtedly, this has more to do with the harsh and enduring economic realities than with some inherent cultural trait. Still, the two factors are not independent, as the former will often shape the latter if given enough continuity in time. Some progress is occurring, as can be discerned from videos describing community “paramedics” (more correctly—volunteers) being trained from among lay persons in rural areas [7].

### Emergency departments

At present, a small number of Western-style emergency departments (ED) exist, including ones at the Dzhanelidze Emergency Care Institute in St. Petersburg, at select hospitals in the republic of Tatarstan and few other locations [8]. Still, within such EDs there remains a tendency for the sickest patients (“red designation”—those needing immediate resuscitation and/or surgical interventions) to be treated by specialists rather than emergency care physicians very early and/or to bypass the geographic ED altogether. While their total number is negligible, viewed against the nation’s territorial spread, these first contemporary-style EDs represent important conceptual and physical constructs integral to the overall strategy of modernizing Russia’s emergency care system.

In the last few years, several publications have surfaced in Russian emergency care journals describing the new conceptual model of the ED. Preliminary data from the abovementioned pilot EDs has also been presented describing specific outcomes sought and monitored, such as the ED’s effect on reducing unjustified hospitalizations [9, 10].

Paradoxically, the idea of the modern ED in terms of its basic mission and functions is not as novel to the Russian medical community as it may appear at first glance.

Since the nineteenth century, the majority of Russian hospitals (or their individual specialty departments at large facilities) have had admission wards (AWs; sometimes translated “receiving bays”) intended for the initial reception, triage (surgical/medical), and sanitary screening of patients. The gradual evolution of such units in Russia has been described in some detail in a recent English language article [11] and will not be repeated here.

It suffices to say that prior to the 1960s, the Russian AWs were not altogether different in terms of physical space and basic capabilities from many ERs of the same era in the USA—for example, the “basement ED,” as described by John McDade on page 35 of Brian Zink’s *Anyone, Anything, Anytime: A History of Emergency Medicine* [12].

In 1963, the USSR Ministry of Health introduced the concept of hospitals of emergency medical care (HEMC), which became the preferred receiving facilities for ambulance traffic in urban and other populated areas. The new requirement for more coordinated emergency care at HEMCs placed new demands on AWs. In response, over the subsequent 20 years and into the 1980s, AWs evolved into what became known as the “receiving diagnostic departments” (RDs) of HEMCs. While not matching all capabilities of a true ED, an HEMC’s RD typically has examination gurneys and procedural areas, a resuscitation “room,” and access to EKG, laboratory services, and radiography.

The old-fashioned AWs (still common at rural and small hospitals) and the more modernized RDs (HEMCs and large central facilities) are the most common “ER” setups found in Russian hospitals today. Thus, while neither type is a full service emergency department, they both fulfill a similar function.

One can thus argue that it is not the ED itself that is truly novel for the Russian healthcare system today but rather the role of the *emergency physician* as a key player within the in-hospital domain of emergency care. In Russia, no such role has existed or entered discussion until most recently.

Attempts to introduce the ED model and the emergency physician role (starting with select locations in large cities) undertaken by the Russian Society of Emergency Medical Care [13] and by few other entities have been met with mixed attitudes, including frank skepticism and resistance [14]. In part, the latter views can be attributed to factors outside of the immediate emergency care reform, e.g., the overall decline of the post-Soviet infrastructure coinciding with the shrinking healthcare workforce due to aging. Other reasons for such reactions stem from difficulties encountered during the ongoing transition to the government’s newly adopted way of financing healthcare (the Mandatory Medical Insurance system). There may also be the perception that many existing physician jobs are being threatened—including positions on the ambulance services.

### Emergency physicians

It is true that historically and until today, in Russia there have never been “emergency physicians” in the Western sense of doctors primarily working at designated emergency departments. What is not true is that no physicians exist whose professional practices are restricted to emergency care.

One of such specialties is *anesthesia*-*reanimation*, better translated as anesthesia-resuscitation. Such providers are involved in both in-hospital and pre-hospital care. In the in-hospital, they run resuscitation departments (ICUs specializing in resuscitation), and in the pre-hospital setting, they are the most common type of a physician in charge of a resuscitation crew (the classic *reanimation* yellow ambulance one may see on the street). Arguably, this cohort of physicians represents one group of existing specialists whose required skills closely match those of emergency physicians.

Second, the often encountered claim regarding “generalists” without specific training working on the majority of ambulance units is not accurate in reference to the three most recent decades. While such a situation existed through the 1970s, one must recall that it was during the same time period that the majority of EDs in countries with the so-called Anglo-American model were also staffed by an incidental assortment of doctors. By 1982, emergency medical care (*skoraya meditzinskaya pomosch*, SMP) in USSR became a certifiable specialty, albeit one constrained to pre-hospital field work [15]. One-year internships in “SMP” and longer clinical *ordinaturas* (residencies) became available to students graduating medical schools as internists, surgeons, or pediatricians (at that time, such basic specialization was granted upon completion of all 6 years of medical education following high school).

Finally, while any point of view is by definition subjective, to the majority of emergency care providers in Russia, including the current career ambulance physicians, the model of emergency care they have inherited and are hoping to improve is not and has never been “Franco-German.” Rather, it is considered to be fully Russia’s own, shaped by the nation’s own medical leaders over the last two centuries, and one that has paid daily tribute to the country’s unique features—even as simple as climate and territory.

### Pre-hospital care

The basics of the European model’s general approach to pre-hospital care, as well as the Soviet two-tier system of ambulances (basic vs. specialized), have been well described elsewhere [1, 16, 17].

However, it is often overlooked that while physicians have always worked and continue to work on the Russian ambulances now, they have never been the predominant ambulance workforce. By some estimates, the Russian midlevel providers, known as *feldshers*, make up as much as 80 % of the crews in total. In remote and rural areas, this percentage may often approach 100 %. This unique profession, tracing its name to German military field medics, is once again at the forefront of pre-hospital emergency care today, as efforts are underway to gradually reduce both the variety of specialized ambulance units and the number of physicians staffing them. Still, challenging questions remain—for instance, what is the continued need for pre-hospital thrombolysis capabilities (and thus, questionably, physician supervision), given that the country’s geography limits access to hospitals in vast areas of land?

Paramedics (implying the established profession and not simply a label for any pre-hospital provider) do not exist nor are they envisioned in Russia as of now. But surrounding issues do arise.

One topic of ongoing debates is the future of Russian ambulance drivers. Traditionally, the drivers have not received any medical training. They were instead responsible for monitoring a vehicle’s operational condition and performed the frequently needed on-the-spot repairs. They also provided navigation skills based on memory and knowledge of the local streets. In the Soviet times the need for a separate driver was dictated, in part, by the fact that relatively few persons owned automobiles and hence held driver’s licenses—almost none among women, who made up (and continue to make up) the majority of the pre-hospital workforce.

In today’s changing environment of car ownership, satellite tracking, and online maps, many have proposed the replacement (or up-training) of the mere driver with a medical person of sorts: a medical technician or perhaps another midlevel provider. Yet, others have pointed out that while professional driving skills are at least helpful in city traffic, in the peripheral areas filled with unlit and unpaved roads such skills remain a necessity.

Overall, in the near future, one is likely to observe a shrinking number of categories of specialized ambulance units [18] and a gradual replacement of ambulance physicians with midlevel providers. Even now, very few basic-level emergency ambulances are routinely staffed by physicians.

### Separation of emergent and urgent ambulance services

In USSR, a multi-specialty ambulatory care center, the *polyclinic*, was assigned to a specific city district or rural area. An average primary care provider, such as a pediatrician, would split their work week between having clinic hours and making house calls within an assigned zone covering several blocks. While in theory many polyclinic services and specialists were available on a first come first serve basis, the “03” ambulance system quickly became overused by the so-called “non-profile” patients (those without emergent conditions). Such patients would primarily rely on the ambulance personnel for their various medical needs [19]. Of note, this trend continues until today, adversely affecting ambulance response times and crew burnout rates.

In order to at least partially compensate for the undue demand on ambulances, walk-in urgent care centers have been set up within the polyclinic structure. More importantly, separate ambulance services termed “urgent” as opposed to “emergent” have been split away from main EMS to augment such centers. Based immediately adjacent to the polyclinics, the urgent ambulances are also staffed by urgent care or primary care personnel. Calls are re-directed to these units by the main dispatch depending on specific criteria. Examples include fever alone or uncontrolled hypertension without other symptoms. Unlike the commonly accepted goal of arrival within 20 min for “03” EMS, response times of up to 2 hours may be acceptable for the urgent units. This separation of the two ambulance services is not new—it has been attempted in the past at various times of USSR history. Proponents and opponents of this dual system continue their debate until today.

Two other types of urgent care facilities have existed traditionally. These are the *travmpunkt* (literally “trauma point”) providing basic minor trauma care services for walk-ins and the rural *feldsher-obstetrical* “point” staffed, as its name implies, by midlevel providers. The future need for these facilities and the way of integrating them into the envisioned new system, one containing modern emergency departments, remain unclear.

### Emergency care professional societies

Several national organizations best described as emergency care professional societies have been created since the turn of the twenty-first century. The National Scientific and Practical Society of Emergency Medical Care, founded at a medical university in Moscow, has worked on cadre development and improvements for the existing system. It has also focused on increasing research efforts and evidence-based practice within EMS, urgent care, and primary care practice settings [20]. Another organization founded more recently and based at the renowned Sklifosovsky Institute of Emergency Care in Moscow has chosen as its mission “uniting [all] specialists (resuscitation specialists, emergency care physicians, surgeons, cardiologists…) working in the area of medicine of emergency conditions” [21]. Finally, the Russian Society of Emergency Medical Care mentioned above is perhaps the most forward organization, attempting to not only improve but also shape the future of emergency care in Russia. All three organizations display their respective journals online, with abstracts available in English.

### Limitations

This brief overview does not aim to be comprehensive or to replace the few works on this topic published in English. Important themes not covered included systems of trauma care, pediatric emergency care, plans for creating or improving inter-facility and inter-regional relationships among entities participating in emergency services, and financial and legislative support of the emergency care system as a whole, among many others. The main goal of this paper has been to expose the reader to select but key issues faced by Russia’s emergency care community at present and to hint at the spectrum of viewpoints which abound.

## Conclusion

Undoubtedly, all of the topics discussed above point to the two main issues at hand. The first has to do with the future well-being of Russia’s emergency care providers—a characteristic directly affecting their ability to consistently carry out their vital role. The second rests on the question—how well will the new system, however constructed, match the real needs of the Russian society at large in terms of its demand for emergency care services?

Presented with a combination of challenges that are both universal and country-specific, the Russian medical community is facing tough choices along the road towards a more modernized healthcare system. Emergency medical care is widely recognized as imperative for the overall health of the nation. The need for a hospital-based physician practice focused solely on emergency care is slowly coming into recognition, immersed in a pool of diverging professional and public opinions held at a time of substantial economic hardships.

In the near future, it would be of interest and value for the world’s emergency medicine community to see more papers by Russian authors come out describing the operation of the planned EDs and the proposed pathways for training (or re-training) the new ED-based emergency care providers. While the Russian emergency care system is likely to take on a unique shape, not exactly matching that in other nations, ultimately its effectiveness will be measured using a number of universally accepted metrics—for instance, any reduction in mortality of victims of motor vehicle accidents.

## Abbreviations

AW: admission ward
ED: emergency department
EMS: emergency medical services
HEMC: hospitals of emergency medical care
ICU: intensive care unit
RD: receiving diagnostic department
